# Salvianic Acid A Sodium Promotes the Recovery of Motor Function After Spinal Cord Injury in Rats by Reducing Microglia Inflammation through Regulating MIP2/Vdac1/Ndufa12 Signaling Axis

**DOI:** 10.1111/os.12808

**Published:** 2020-10-20

**Authors:** Liping Li, Yuanyuan Liu, Xia Zhao, Chao Qi, Yi Zhang, Yingze Zhang, Tengbo Yu

**Affiliations:** ^1^ Department of Orthopaedic Surgery The Affiliated Central Hospital of Qingdao University Qingdao China; ^2^ Department of Orthopaedic Surgery The Second Clinical Medical College of Qingdao University Qingdao China; ^3^ Department of Oncology The Affiliated Central Hospital of Qingdao University Qingdao China; ^4^ Department of Oncology The Second Clinical Medical College of Qingdao University Qingdao China; ^5^ Department of Orthopaedic Surgery Affiliated Hospital of Qingdao University Qingdao China; ^6^ Department of Orthopaedic Surgery The Third Hospital of Hebei Medical University Shijiazhuang China

**Keywords:** Microglia, Motor function, SAAS, SCI

## Abstract

**Objective:**

To clarify the effects on and the mechanism of salvianic acid A sodium (SAAS) in the recovery of motor function after spinal cord injury.

**Methods:**

*In vivo* and *in vitro* experiments were carried out in this research to determine the effects of SAAS on tissue damage, neuron survival, microglia polarization, and inflammation after spinal cord injury (SCI). Differentially expressed genes treated with SAAS were screened by transcriptome sequencing, and the molecular mechanism was investigated simultaneously.

**Results:**

The results revealed that SAAS could promote type M2 polarization of microglia and reduce the proportion of type M1. In this way, it reduced the secretion and expression of inflammatory factors. Compared with Lipopolysaccharides(LPS), 345 genes were upregulated and 407 genes were downregulated in the LPS + SAAS treatment group. In the SAAS group, expression levels of Ndufa12, IL‐6, TNF‐α, and Vdac1 were significantly reduced, while a marked elevation was found in MIP2. In addition, results found in an animal model showed that SAAS could obviously facilitate motor function recovery of mice after spinal cord injury, and it had a good protective effect on spinal cord tissue and neuron cells.

**Conclusion:**

As a result, the present study clarified both the protective effect of SAAS on neurons after spinal cord injury and the anti‐inflammatory effect of microglia, which is expected to serve as a theoretical basis for clinical treatment.

## Graphical abstract

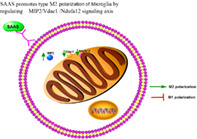

Salvianic acid A sodium can improve the recovery of motor function in rats with spinal cord injury. Its mechanism may be to protect neurons and promote type M2 polarization of microglia by regulating the MIP2/Vdac1 /Ndufa12 signaling axis.

## Introduction

Once the central nervous system (CNS) is injured, nerve axons do not easily regenerate, so nerve function may not fully return. This remains an unsolved problem of neuromedicine^1^. Microglia are derived from bone marrow mononuclear cells. They account for approximately 5% of total gliocytes in the CNS^2^, ^3^. When the CNS is injured, microglia are activated early. Some scholars have pointed out that once the CNS is injured, microglia activation and neuron apoptosis occur simultanously^4^, ^5^. This finding indicates that the process of CNS injury is accompanied by the activation of microglia. As a result, the activation and proliferation of microglia is the first glial reaction after CNS injury. Some research shows that macrophages marked with chondroitin sulfate proteoglycan can be found 24 h after injury. On microscopic observation, fully activated microglia were displayed in various shapes, including circular, amoebiform, and rod‐shaped, while partially activated microglia showed branching shapes^6^, ^7^, ^8^. In addition, damage was accompanied by obvious activation of astrocytes and reactive gliosis, featuring coarseness of protuberances, hypertrophy of cell bodies, and upregulation of the expression of intermediate fibers. Eventually, dense glial scar formation came into being. It has been proved that culture medium of microglia contribute to astrocytes proliferation. *In vivo*, the activation of astrocytes and the expression of glial fibrillary acidic protein (GFAP) were closely related to microglia‐derived factors, indicating that microglia were involved glial scar formation^9^, ^10^. In addition to glial cells, glial scars also contain a variety of extracellular substrate, including IL6, which is harmful to axon growth^10^, ^11^. Moreover, in some relevant study, the glial scar of the injured part of the spinal cord was excised in rats and no obvious growth of axon was observed, suggesting that the development of scar formation is a complex process of biological regulation^12^, ^13^. Further understanding the mechanism and developing new therapeutic methods are critical.

Through obviously reducing the area of cerebral infarction, SAAS improves the neurological deficit and decreases brain water content^14^. *Salvia miltiorrhiza* injection can obviously decrease pathological changes both in brain index and water content caused by acute cerebral ischemia in rats, reduce the permeability of capillaries in the brain, and maintain normal morphology and structure of nerve cells in brain tissue^15^, ^16^. SAAS could prevent calcium influx and significantly inhibited prostaglandin E2 and TXB2 produced by peritoneal macrophages in rats^17^, ^18^. The regulation of SAAS in the production of cytokines by mononuclear macrophages was observed *in vitro*. The results showed that SAAS could activate mononuclear macrophages to secrete TNF α, IL1, IL6, and IL‐8, while the quantity of secretion was less than that of endotoxin‐stimulated mononuclear macrophages^19^, ^20^. Clinical application of SAAS in patients suffering from septic shock is expected to improve survival rates. In this study, *in vivo* and *in vitro* tests were conducted to determine the effects of SAAS on tissue injury, neuron survival, microglia cell polarization, and inflammation after SCI. The molecular mechanism was also investigated. As a result, this study is expected to provide a therapeutic option and theoretical basis for clinical treatment.

## Materials and Method

### *Cell Culture and Treatment*

BV2 cells were purchased from Procell Life Science & Technology. The BV‐2 cells were cultivated in Dulbecco's modified Eagle medium, supplemented with 10% fetal bovine serum, 100 units/mL penicillin, and 100 μg/mL streptomycin. Cells were incubated at 37°C in a humidified atmosphere containing 5% CO_2_ and passaged every 2 days. The BV2 cells were inoculated in six‐well plates. Inoculation density was 10^5^/mL, with a volume of 2 mL per well.

The cells were classified into three groups: an LPS group, an LPS + SAAS group, and a control group. There were three multiple pores in each group. For the LPS group, the culture medium was changed 12 h after cell inoculation. The medium was removed, followed by culturing for another 12 h. The cells were gently rinsed three times using PBS, then culture medium containing 1 ug/mL LPS was added and cells were cultured for 12 h. In the LPS + SAAS group, the SAAS culture medium with a concentration of 40 ug/mL was added after 12 h of cell inoculation. The medium was removed, followed by culturing for 12 h. The cells were gently rinsed three times using PBS, then culture medium containing 1 ug/mL LPS was added and cells were cultured for 12 h. For the control group, the culture medium was changed 12 h after cell inoculation. The medium was removed, followed by culturing for 12 h. The cells were gently rinsed three times using PBS, then culture medium was added and cells were cultured for 12 h. Finally, the cells were collected for testing.

### *Immunofluorescence Assay*

Tissue sections were fixed with 4% paraformaldehyde at room temperature for 15 min. The tissue sections were washed and rinsed with PBS three times, for 3 min each time. Most of the residual PBS was removed with absorbent paper. The tissue sections were permeated with 0.5% Tritonx‐100 for 15 min. Next, 6% normal goat serum was added to the tissue sections, which were sealed at room temperature for 30 min. The sealing liquid was drained with absorbent paper. Adequate diluted primary antibody (dilution ratio: 1:100) was added directly to each section, which were incubated in a box containing wet tissue paper at 4°C overnight. The sections were taken out of the box containing wet tissue paper and maintained at a temperature of 37°C for 45 min; then the tissue sections were washed and rinsed with 1 × PBS three times, for 3 min each time. After removing most residual PBS solution on the tissue sections with absorbent paper, diluted fluorescence secondary antibody Anti‐IgGCy3 was added (dilution ratio: 1:100), and the tissue sections were incubated in a box containing wet tissue paper at room temperature for 1.5 h. Following the addition of fluorescence secondary antibody, all the subsequent steps should be carried out in a dark room if possible. The tissue sections were washed and rinsed with PBS three times, again for 3 min each time. DAPI was added, the tissue sections were incubated in the dark for 5 min, and the specimens were nucleated. Then the tissue sections were washed and rinsed with 1× PBS three times, for 3 min each time. Remaining DAPI was absorbed. The residual PBS was removed from the tissue sections with absorbent paper, and the sections were sealed with anti‐fluorescence quencher; then the images obtained were observed with a fluorescence microscope.

### *Nissl Staining*

The specimens collected were cleaned in PBS and fixed with 4% paraformaldehyde. The tissues were hydrated in 75% ethanol, 85% ethanol, 95% ethanol I, 95% ethanol II, 100% ethanol I, and 100% ethanol II for 2 h each. The dehydrated tissues were soaked in 1/2 xylene, xylene I, and xylene II for 10 min. The transparent treated tissue was immersed in melted paraffin for 3 h. The tissue in the paraffin block was cut into sections of 5‐um thickness with a slicer and spread on an adherent glass. The sections were placed in a baking machine at 55°C, making sure that the tissue sections stuck tightly to the slide glass. The paraffin sections were soaked in xylene I, xylene II, and 1/2 xylene II for 10 min each. The dewaxed paraffin sections were soaked in 100% ethanol I, 100% ethanol II, 95% ethanol I, 95% ethanol II, 85% ethanol, and 75% ethanol for 5 min each. The sections were then rinsed with double distilled water three times, for 2 min each time. Toluidine blue stain was added to the sections, and a dyeing cylinder was placed in an incubator at 60°C for 45 min. The sections were rinsed with distilled water before washing with 70% ethanol. The sections were differentiated in 95% ethanol rapidly. Anhydrous ethanol was quickly dehydrated. The sections were treated with xylene and sealed with neutral resin.

### *Western Blot*

The glue was taken from the refrigerator (at 4°C) and placed in an electrophoresis tank. Then, 500 μg of total proteins were taken from each specimen and mixed with 5× SDS specimen buffer at a ratio of 4:1. After mixing, the concentration of the proteins was approximately 3.3 μg/μL. The proteins were denatured after being boiled for 5 min. Denatured total proteins of 60 μg were selected for specimen loading. After the spacer gel was electrophoresed with a voltage of 80 v, the voltage was converted to 120 V, before waiting until the bromophenol blue reached the bottom of the adhesive plate without exceeding the bottom of the adhesive plate. The clip was loosened to keep the black side horizontal, and the sponge pad, filter paper, glue, PVDF film (activated by methanol), and filter paper were placed on the black side of the clip, in turn. At the same time, the electrophoretic fluid was replaced with transfer fluid. The current was switched to a constant current of 300 mA and the duration of the transfer was 60–90 min. The film was taken out, marking both the front and back side, and washed with TBST for 1 min. After that, the membrane was sealed with 5% skim milk for 1 h at room temperature. Followed sealing, it was washed with TBST three times, for 5 min each time. The primary antibody solution was diluted with 3% BSA at a ratio of 1:1000–2000. The membrane was incubated overnight at 4°C. Next, the membrane was rinsed with TBST three times, for 10 min each time. The secondary antibody was diluted to a certain concentration (1:1000) with blocking buffer and incubated at room temperature for 1.0 h. The membrane was rinse with TBST three times, for 10 min each time. The ECL exposure solution was mixed well according to the ratio of solution A and solution B (1:1) and evenly spread over the entire film. After 1 min reaction, the membrane was placed into an exposure meter for exposure detection.

### *Quantitative PCR*

Reverse transcription was performed after RNA extraction, and procedures were conducted according to the instructions for the Promega reverse transcription kit. Then, 4‐μL RNA templates were taken for reverse transcription reaction; the instrument used was ABI9700 (ABI, USA). The reaction system was as follows: 5× reverse transcription buffer, 4 μL; arbitrary primers, 0.4 μL; deoxynucleoside triphosphates (10 mM), 0.5 μL; MMLV (U/μL), 1 μL; diethyl pyrocarbonate (DEPC) water, 10.1 μL; and RNA template, 4 μL. Reaction condition: 37°C1h, then 95°C for 3 min. According to the instructions for using the Promega PCR mix, the reaction system was configured as follows: 2× PCR mix buffer, 10 μL; upstream primers, 1 μL; downstream primers, 1 μL; template, 2 μL; and DEPC water, 6 μL. Reaction condition: 93°C for 3 min, then 93°C for 30 s, and 55°C for 45 s, for a total of 40 cycles, at 72°C for 5 min, and 4°C thereafter.

### *Cell Phenotype Identification by Flow Cytometry*

A total or 1 × 10^5^ cells were prepared, digested by trypsin, then centrifuged at 170 ×g for 10 min, and rinsed twice with PBS. PBS resuspended cells were loaded into Eppendorf (1 mL/ tube) separately. The cells were centrifuged at 680 ×g for a further 6 min. The supernatant was discarded, and dispatch cells and antibodies were added, respectively (ARG‐1,1:500; CD206 1:500; CD86, 1:500; and CD80, 1:500). Cells were incubated for 30 min at 4°C away from light. Then, 1 mL PBS was added to each tube with centrifugation at 680 ×g for 6 min, and repeat twice. Finally, after adding 0.5 mL PBS to re‐suspend the cells, the cells were removed by membrane filtration to avoid clogging the flow meter.

### *Animal Model Preparation*

Male Sprague–Dawley rats (200–250 g) were purchased from Pengyue Laboratory Animal Company in Jinan, China. Mice were housed in a 12:12 h light–dark cycle at a temperature of 23–25°C with a routine diet and water available. The experiment was carried out 1 week after adaptive feeding. For anesthesia, 7% chloral hydrate (0.5 mL/100 g) was intraperitoneal injected into the rats, and a surgical plate was fixed in prone position. A 4‐cm longitudinal skin incision with a specific diet and water availablen was made with T10 as the center to expose the T10 vertebrae and the vertebral plate. The T10 vertebrae, the interspinous ligament, the yellow ligament, and the vertebral plate were exposed with hemostatic forceps, and both the endorhachis and the spinal cord corresponding to T10 were exposed. A self‐made controllable percussion instrument (composed of cuff, 10 g weight article, impact bar, scabbard, and stereoscopic locator) was used to bump the impact bar, with a 10‐g weight article falling freely from a height of 10 cm. The impact bar was connected to the exposed T10 spinal cord. The posterior median sulcus of the spinal cord was impacted. The spinal cord was compressed by the impact bar for 2 min and then sutured layer by layer. Postoperatively, rats were given penicillin injections for 3 days to prevent infection. The rats urinated with an artificial bladder three times a day until reflex bladder emptying was established. With the dosage at 20 mg/kg, SAAS was prepared into a solution with a concentration of 5 mg/mL and intraperitoneally injected at 4 mL/kg body weight. Specimens were collected after 10 days of continuous injection.

In each Sprague–Dawley group, 7% chloral hydrate (0.5 mL/100 g) anesthesia was performed. After successful anesthesia, the spine was taken out. Excess muscle and other tissues were removed, and the spinal column near the T10 spinal cord was directly fixed with 4% paraformaldehyde. After fixation for 72 h, the spinal cord was carefully removed for subsequent analysis. The other part was taken out of the spinal column and stripped directly from the spinal cord, and stored at −80°C for subsequent analysis.

A total of 51 rats were randomly divided into three groups: a sham group, an SCI + SAAS group, and an SCI + saline group (*n* = 17). In the sham group, only the T10 spinal cord was exposed and no other treatment was performed. In the SCI + SAAS group and the SCI + saline group, T10 spinal cord injury models were made according to the method described above. In the SCI + SAAS group, the rats were immediately intraperitoneal injected with SAAS (20 mg/kg) after the operation and then intraperitoneally once a day until the animals were killed. The sham group and the SCI + saline group were injected with an equivalent dose of normal saline.

Spinal cord tissues for western blot (*n* = 3 per group) and quantitative PCR (qPCR) (n = 3 per group) detection were collected 3 days postoperation. Spinal cord tissues used for immunofluorescence and hematoxylin and eosin (HE) staining (n = 3 per group) were collected 1 week after the operation and made into longitudinal sections (5‐μm thick), while spinal cord tissues for Nissl staining (n = 3 per group) were collected 1 week after the operation and made into transverse sections (5‐μm thick). The remaining five rats in each group were used for Basso, Beattie and Bresnahan (BBB) score and inclined plate tests. They were killed 4 weeks after the operation.

### *Rivlin Inclined Plane Test and Basso, Beattie and Bresnahan Score Test*

A 5‐mm‐thick rubber pad was placed on the inclined plate in the direction perpendicular to the longitudinal axis of the inclined plate and the axis of the rat body. The angle between the inclined plate and the horizontal plane was gradually increased until the rat could just stay on the plate for 5 s, and the angle was recorded. Each rat was measured three times. The test was repeated three times for each rat and the mean value was determined. The BBB score of the rats was established by two researchers who did not know the animal group, and the mean value was determined.

### *Statistical Methods*

GraphpadPrism5.0 software was used for statistical analysis. The measurement data was expressed as mean ± standard deviation, and the normality test and the homogeneity test of variance were performed on each group. Once the data conformed to the normal distribution with homogeneity of variance, the Tukey test was conducted for comparison between the two groups. The calculation was statistically described by frequency, and the χ^2^‐test was applied for comparison between the two groups. *P* < 0.05 was considered statistically significant.

## Results

### *Effects of Salvianic Acid A Sodium on Polarization of Lipopolysaccharides* *(LPS)‐Induced Microglia*

**Fig. 1:**
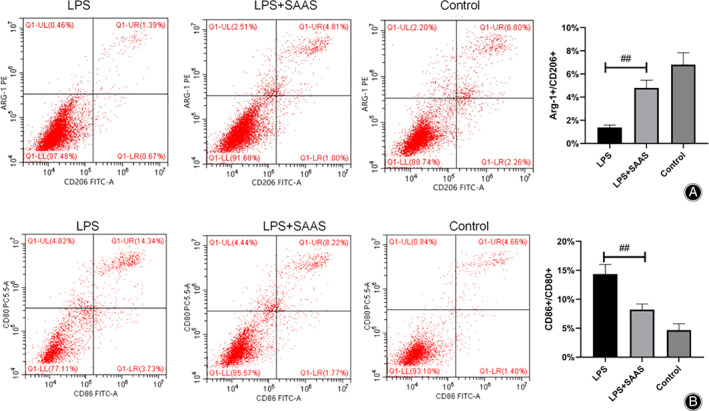
Effects of salvianic acid A sodium (SAAS) on polarization of LPS‐induced microglia. (A) Percentage of Arg‐1^+^/CD206^+^ cells. (B) CD86^+^/CD80^+^ cell identification. Compared with the control group, the number of Arg‐1 ^+^/CD86^+^ cells decreased and the number of CD86^+^/CD80^+^ cells increased after LPS treatment. After SAAS was initiated, the number of Arg‐1 ^+^/CD86^+^ cells was elevated and the number of CD86^+^/CD80^+^ cells decreased.

To clarify the effect of SAAS on LPS‐induced BV‐2 inflammations, flow cytometry was used to determine the polarization of BV‐2 cells after *in vitro* culture of BV2 cells with Lipopolysaccharides (LPS), LPS + SAAS, and equal volume of culture medium. The results showed that compared with the control group, the number of Arg‐1 ^+^/CD86^+^ cells decreased and the number of CD86^+^/CD80^+^ cells increased after LPS treatment. After SAAS was initiated, the number of Arg‐1 ^+^/CD86^+^ cells was elevated and the number of CD86^+^/CD80^+^ cells decreased (Fig. 1A,B). This revealed that LPS could promote M1‐type polarization of microglia and reduce the M2‐type polarization ratio. However, SAAS could promote M2‐type polarization of microglia and reduce the proportion of M1‐type SAAS, thus reduce the secretion and expression of inflammatory factors.

### *Differential Expression Genes After Salvianic Acid A Sodium Treatment*

**Fig. 2:**
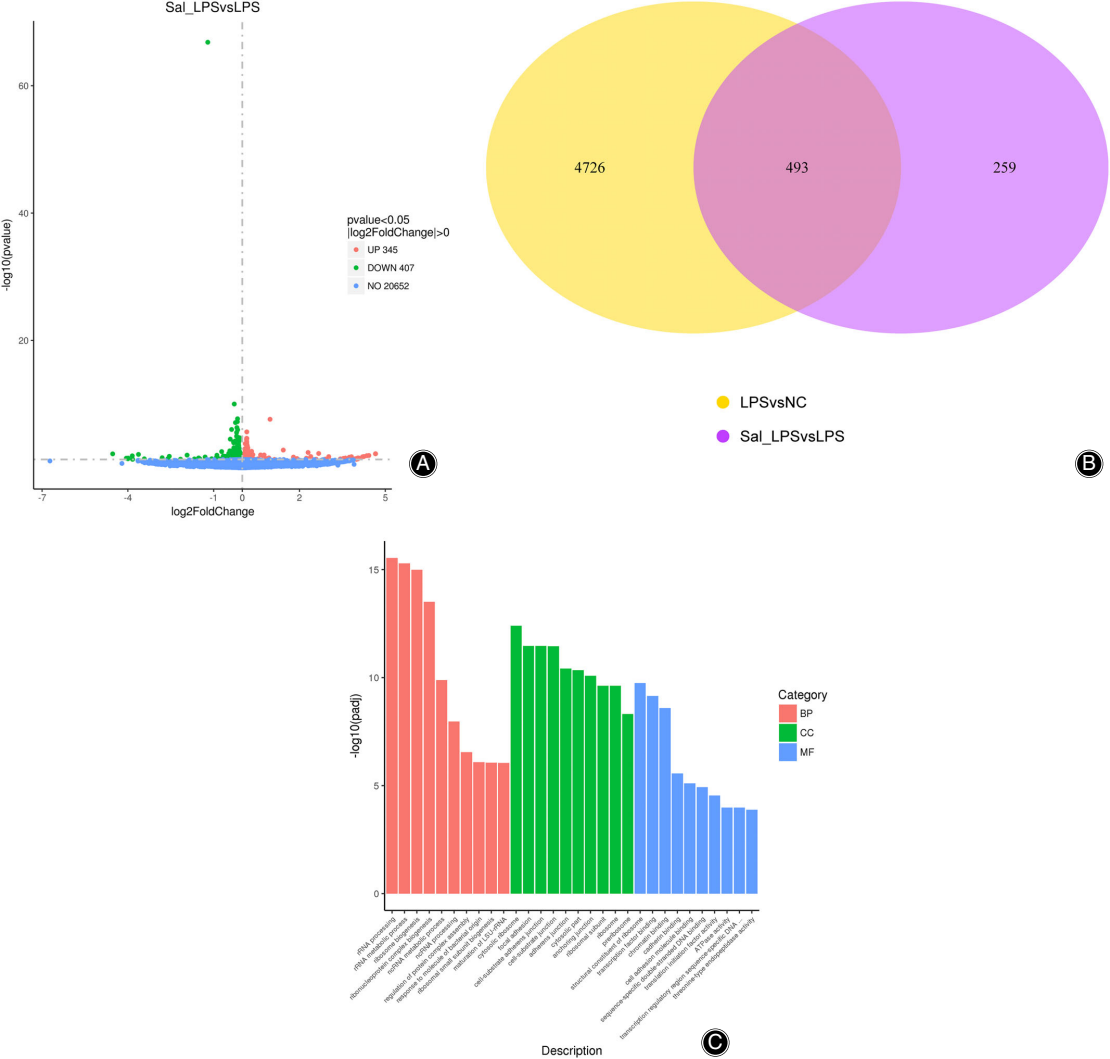
Differential expression genes identified by transcriptome sequencing after salvianic acid A sodium (SAAS) treatment. (A) Volcanic map shows the distribution of differential genes. (B) Venn diagram shows the overlap of differential genes among different comparison combinations. The sum of all numbers circled in the Venn diagram represents the total number of the differential genes in the comparison combination, and the overlapped area represents the shared number of differential genes of the combinations. (C) GO analysis. (D) Sequencing results of quantitative PCR verification. (E) Differential gene expression of the protein by western blot verification. The expression levels of Ndufa12, IL‐6, TNF‐α, and Vdac1 were largely elevated, while a marked decrease was found in MIP2 after LPS treatment.

Three groups of cells were collected for transcriptome sequencing. The results demonstrated that, compared with the control group, expression levels of 2,785 genes were significantly upregulated while expression levels of 2,434 genes were greatly downregulated after Lipopolysaccharides (LPS) addition. Compared with LPS, 345 genes were upregulated and 407 genes downregulated in the LPS + SAAS treatment group. Through analysis, it was found that there were 493 shared differentially expressed genes in the LPS *vs* NC group and the LPS + SAAS *vs* LPS group (Fig. 2A–C). Among them, expression levels of Ndufa12, IL‐6, TNF‐α, and Vdac1 were significantly elevated, while a marked decrease was found in MIP2. The expression increase caused by LPS was suppressed in the LPS + SAAS group. The detection results for both qPCR and western blot were consistent with the sequencing results. The expression levels of Ndufa12, IL‐6, TNF‐α, and Vdac1 were largely elevated, while a marked decrease was found in MIP2 after LPS treatment (Fig. 2D,E). By adding SAAS, the LPS‐induced increase in gene expression could be significantly recovered.

### *Animal Model Preparation and Postoperative Scores*

**Fig. 3:**
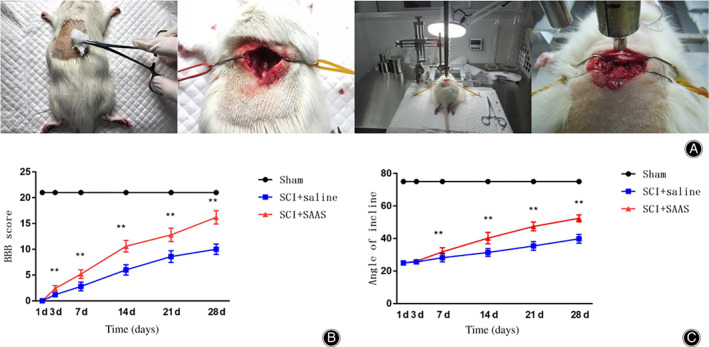
Animal model preparation and postoperative scores. (A) Animal model preparation process: We established spinal cord injury models at T10 level using the weight drop method (10 g*10 cm). (B) Basso, Beattie and Bresnahan (BBB) scores. (C) Statistical results of inclined plate test. The salvianic acid A sodium (SAAS) treatment group revealed significantly greater functional recovery than for the SCI + saline group. SCI, spinal cord injury.

Subsequently, we examined the impact of SAAS on both SCI motor function recovery and neuron survival in an animal model (Fig. 3A). The BBB score and improved Rivlin inclined plane tests were conducted to evaluate hind limb function in rats of each group. Our BBB score and angle of inclined plane tests showed that the SAAS treatment group experienced significantly greater functional recovery than other groups, indicating that SAAS can improve motor functional recovery after SCI (Fig. 3B,C).

### *Mechanism of Salvianic Acid A Sodium in Spinal Cord Protection*

**Fig. 4:**
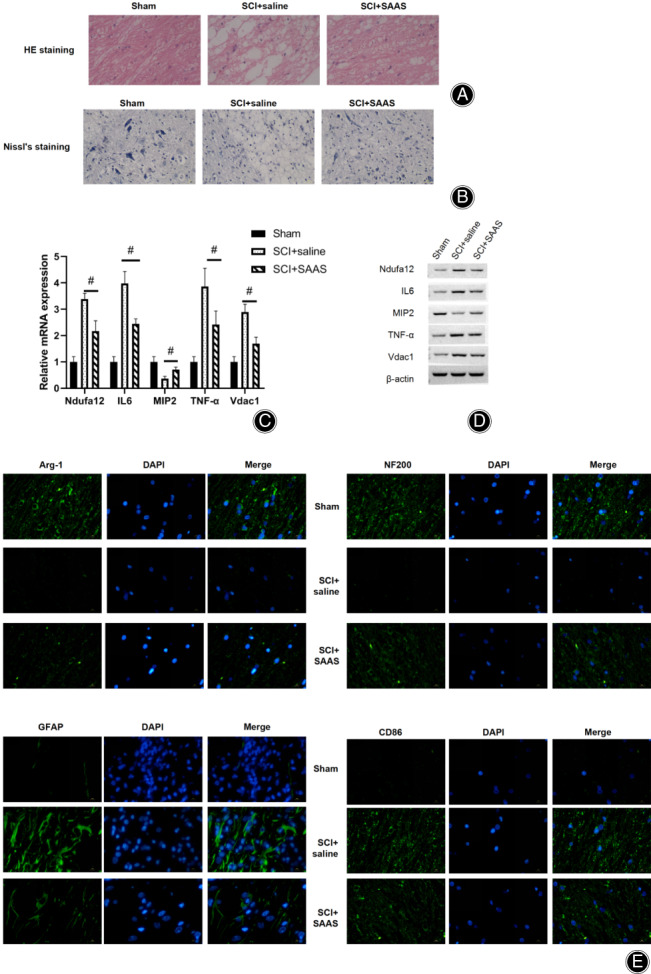
Study on the mechanism of salvianic acid A sodium (SAAS) in spinal cord protection. (A) Hematoxylin and eosin (HE) staining results (40×, 1 week postoperation). In the SCI + saline group, the morphology and structure of the spinal cord were destroyed, with a spacious gap and injuries exhibited. SAAS treatment can reduce the gap and alleviate spinal cord injury. (B) Nissl staining results (20×, 1 week postoperation). Nissl staining suggested that after SCI treatment, the number of neurons decreased, the number of interstitial tissues increased, and the density of tissues was destroyed, while SAAS treatment could effectively alleviate the damage of SCI and protect spinal cord tissues and neurons. (C) Quantiative PCR (qPCR) verification results (3 days postoperation). (D) Western blot verification results (3 days postoperation). The expression levels of Ndufa12, IL‐6, TNF‐α, and Vdac1 were significantly increased, while a marked decrease was found in MIP2 after SCI treatment, verified through qPCR and western blot analysis. By introducing SAAS, the SCI‐induced increase in gene expression could be significantly recovered. E. Immunofluorescence assay results (1 week postoperation). Compared with the SCI + saline group, the expression of Arg‐1 and NF200 dropped, and the expression of GFAP and CD86 increased in the SCI + SAAS group. SCI, spinal cord injury.

Hematoxylin and eosin staining was adopted to observe the structure of the spinal cord. After SCI treatment, the morphology and structure of the spinal cord were damaged, with a spacious gap and injuries exhibited. In the SAAS treatment group, a reduced gap was noticed and the structure of damage was significantly less than that of the SCI group (Fig. 4A). Nissl staining suggested that after SCI treatment, the number of neurons decreased, the number of interstitial tissues increased, and the density of tissues was decreased (Fig. 4B). Similarly, SAAS treatment could effectively alleviate the damage of SCI and protect spinal cord tissues and neurons. Immunofluorescence assay findings revealed that after SAAS treatment, the expression of Arg‐1 and NF200 decreased, and the expression of GFAP and CD86 increased (Fig. 4E). The expression levels of Ndufa12, IL‐6, TNF‐α, and Vdac1 were significantly increased, while a marked decrease was found in MIP2 after SCI treatment, as verified through qPCR and western blot analysis (Fig. 4C,D). By introducing SAAS, the SCI‐induced increase in gene expression could be significantly recovered.

## Discussion

**Fig. 5:**
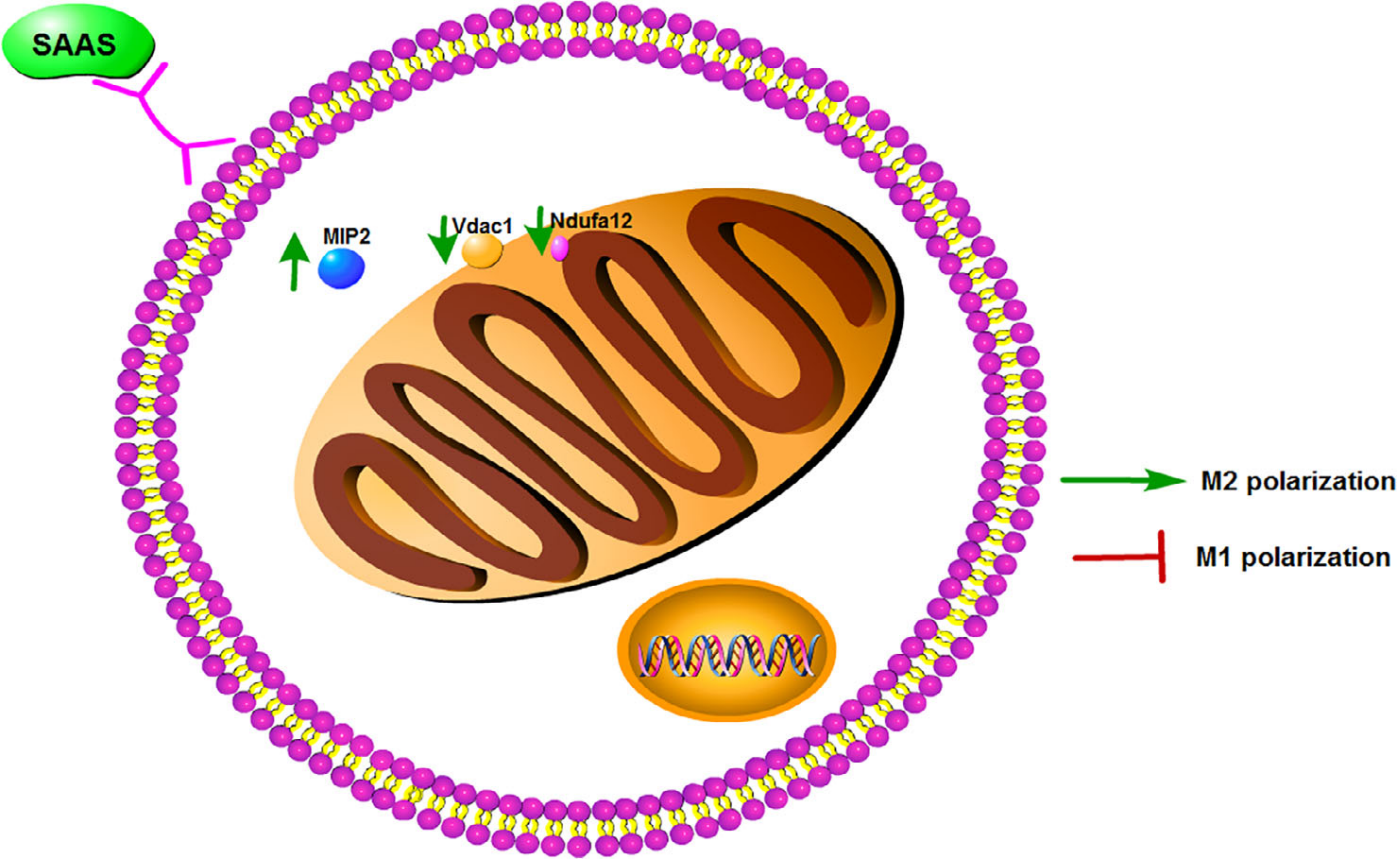
Salvianic acid A sodium (SAAS) promotes type M2 polarization of microglia by regulating the MIP2/Vdac1 /Ndufa12 signaling axis.

As a traditional Chinese herbal medicine in China, *Salvia miltiorrhiza* has diverse uses, including in antibacterial, antiviral, antioxidant, antithrombotic, and antidepressant therapies. SAAS could repair tissue damage and downregulate the expression levels of HIF‐1α, PCNA, and bcl‐2^21^, ^22^. Meanwhile, SAAS was effective in preventing heart damage and improving heart function. SAAS was also found to be involved in actin cytoskeletons, phagosomes, adherent plaques, tight junctions, and apoptosis^22^. The direct binding proteins of SAAS were identified by proteome chip, and a total of 370 proteins were identified as specifically binding to SAAS and were significantly enriched in the metabolic pathway by UPLC‐QTOF‐MS. Twenty‐six potential biomarkers were analyzed and determined with serum metabolomics in rats, including various glycerolipids and a series of aliphatic acids^23^. In this study, we found that SAAS protected spinal cord tissue structure and neurons. Based on BBB score and oblique plate tests, SAAS could protect the motor function of mice with spinal cord injuries (Fig. 5).

Treatment with SAAS was proven to be a safe practice, mainly in phase II metabolism, especially excretion in urine. The pharmacokinetics of SAAS after intravenous administration was evaluated in rats at doses of 15 mg/kg, 30 mg/kg, and 60 mg/kg, respectively. After intravenous administration, SAAS presented linear pharmacokinetics in the dose range of 15–60 mg/kg. The absolute bioavailability of SAAS was 13.72%. Kidney was the major distributed tissue, while there were distributions observed in lung, stomach, muscle, uterus, and heart as well. Within 96 h after intravenous administration, 46.99% of the mother drug was excreted as urine, and 1.16% of the the mother drug was excreted as feces. The 24‐h biliary tract excretion rate of SAAS was approximately 0.83%, and the urinary metabolites were methylated, sulfated, and acetylated, respectively^24^, ^25^. SAAS revealed poor oral bioavailability and could be developed as an injection. In this study, the protective effect of SAAS on the spinal cord and neuron cells was observed by HE staining and Nissl staining after injection in animal model.

MIP2 is located in mitochondria, one of the WDR26 subtypes, and protects cells from oxidative stress. The co‐IP assay was used to isolate the MIP2 protein complex from the rat heart, and mass spectrometry analysis showed that there was an interaction between Vdac1 and MIP2^26^. Overexpression of MIP2 reduced the increase and cell damage of HO‐induced Vdac1, while the loss of MIP2 aggravated the increase and cell damage of HO‐treated H9c2 cells^26^, ^27^. The protective effect of MIP2 on antioxidant stress of cardiomyocytes was partly related to the interaction with Vdac1, thus inhibiting its expression. In addition, Vdac1was closely related to Alzheimer's disease. Decreased Vdac1 expression was beneficial to synaptic activity and improved function, and could alleviate Alzheimer's disease^28^, ^29^. Vdac is located in the outer membrane of mitochondria and acts as a gatekeeper for metabolites and ion exchange between cytoplasm and mitochondria; it is is responsible for regulating mitochondrial phagocytosis and apoptosis^30^. This study found that SAAS could significantly reduce the expression of Ndufa12, IL‐6, TNF‐α, and Vdac1, and significantly increase the expression of MIP2. Combined with previous research results, we speculate that SAAS may protect nerve cells and the spinal cord from LPS‐induced inflammation through the mitochondrial antioxidant defense system, thus promoting the recovery of motor function after spinal cord injury. Further research is suggested to explore this consideration.

### *Conclusion*

Salvianic acid A sodium could obviously restore the motor function of rats after spinal cord injury, and produced a good protective effect on spinal cord tissue and neuron cells. As a result, this study clarified both the protective effect of SAAS on neurons after spinal cord injury and the anti‐inflammatory effect of microglia, which is expected to provide a theoretical basis for clinical treatment.
